# Draft Genome Sequence of Mycobacterium virginiense Strain GF75, Isolated from the Mud of a Swine Farm in Japan

**DOI:** 10.1128/genomeA.00362-18

**Published:** 2018-04-26

**Authors:** Toshihiro Ito, Fumito Maruyama, Kotaro Sawai, Keiko Nozaki, Keiko Otsu, Kenji Ohya

**Affiliations:** a Department of Microbiology, Graduate School of Medicine, Kyoto University, Kyoto, Japan; b Laboratory of Veterinary Microbiology, Faculty of Applied Biological Sciences, Gifu University, Gifu, Japan; c Gifu Prefectural Chuo Livestock Hygiene Service Center, Gifu, Japan; d United Graduate School of Veterinary Sciences, Gifu University, Gifu, Japan

## Abstract

Mycobacterium virginiense, a newly described species of the Mycobacterium terrae complex, is a cause of tenosynovitis and osteomyelitis in the United States. Here, we report the 4,849,424-bp draft genome sequence of M. virginiense strain GF75, isolated from a mud sample taken from a Japanese swine farm.

## GENOME ANNOUNCEMENT

Nontuberculous mycobacteria (NTM), including the Mycobacterium terrae complex, are environmentally transmitted pathogens that are relevant to human and animal health (1). A newly described species of the M. terrae complex, M. virginiense, was recently recognized as an infectious agent of clinical importance (2). M. virginiense was first described in 2016 on the basis of three independent clinical strains as the etiological agent of tenosynovitis and osteomyelitis in the United States (3, 4). The three isolates of M. virginiense in previous studies were acid fast and nonpigmented on Middlebrook 7H10 agar, slow growing, and resistant to various antibiotics, including rifampin and the quinolones (5). The distribution and incidence of NTM are known to vary temporally and geographically (6, 7). Hence, the characterization of NTM, such as M. virginiense, isolated from different sources and/or geographic areas will enable a better understanding of their ecology and epidemiology.

Here, we report the draft genome sequence of an M. virginiense strain isolated from a mud sample taken from a swine farm in the Tokai area of Japan, where an outbreak of swine mycobacteriosis occurred (data not shown). The mud sample was decontaminated overnight with an equal volume of 2% NaOH. The suspension was washed with phosphate-buffered saline (PBS) for neutralization and then inoculated on 2% Ogawa slant (Kyokuto Pharmaceutical, Tokyo, Japan) at 37°C for 4 weeks. Colonies were subcultured on Middlebrook 7H11 agar supplemented with 10% oleic acid-albumin-dextrose-catalase (OADC) enrichment (Becton, Dickinson, MD, USA). DNA was extracted using the PureLink genomic DNA extraction kit according to the manufacturer’s instructions (Invitrogen, MA, USA), and paired-end libraries with an average insert size of 350 bp were prepared. These underwent 2 × 150-bp sequencing on a HiSeq X Ten sequencing platform (Illumina, San Diego, CA, USA) at the Beijing Genomics Institute (Shenzhen, China).

Raw reads were preprocessed using SOAPnuke (8) and assembled using the A5-miseq pipeline (9). Contigs were binned and clustered on the basis of tetranucleotide frequency, G+C content, and differential coverage using BinSanity (10). Reads mapping to the resulting bin were extracted using Burrows-Wheeler Aligner (BWA) (11) and SAMtools (12). Reassembly and final scaffolding were performed using the A5 MiSeq pipeline (9). The resulting scaffold was refined to reduce contamination on the basis of tetranucleotide frequency, G+C content, and differential coverage using RefineM (13). CheckM (14) reported 100% completeness with 1.15% potential contamination for the final draft genome sequence, which was then annotated using the NCBI Prokaryotic Genome Annotation Pipeline (PGAP) (15).

The combined length of the final draft genome sequence was 4,849,424 bp comprising 128 scaffolds with a 67.25% G+C content, a total of 4,714 coding sequences, and 54 predicted RNAs, including 4 rRNAs, 47 tRNAs, and 3 noncoding RNAs. Based on blastn analysis against the NCBI nucleotide database (downloaded on 16 February 2018), 65% and 34% of the draft genome sequence exhibited significant sequence similarity with M. terrae strain NCTC10856 (GenBank accession number LT906469) and M. sinense strain JDM601 (GenBank accession number CP002329), respectively.

### Accession number(s).

The draft genome sequence of M. virginiense strain GF75 has been deposited at DDBJ/ENA/GenBank under the accession number PUEV00000000.
